# Ethnic Differences on Cardiac Rhythms and Autonomic Nervous System Responses During a High-Altitude Trek: A Pilot Study Comparing Italian Trekkers to Nepalese Porters

**DOI:** 10.3389/fphys.2021.709451

**Published:** 2021-08-23

**Authors:** Vittore Verratti, Alessandro Tonacci, Danilo Bondi, Annalisa Chiavaroli, Claudio Ferrante, Luigi Brunetti, Antonio Crisafulli, Paolo Cerretelli

**Affiliations:** ^1^Department of Psychological, Health and Territorial Sciences, University “G. d'Annunzio” of Chieti-Pescara, Chieti, Italy; ^2^Institute of Clinical Physiology, National Research Council of Italy, Pisa, Italy; ^3^Department of Neuroscience, Imaging and Clinical Sciences, University “G. d'Annunzio” of Chieti-Pescara, Chieti, Italy; ^4^Department of Pharmacy, University “G. d'Annunzio” of Chieti-Pescara, Chieti, Italy; ^5^Department of Medical Science and Public Health, University of Cagliari, Cagliari, Italy; ^6^Institute of Bioimaging and Molecular Physiology, National Research Council of Italy, Segrate, Italy

**Keywords:** heart rate variability, urinary catecholamines, himalayas, altitude hypoxia, adaptiveness, blood pressure

## Abstract

Altitude hypoxia exposure results in increased sympathetic activity and heart rate due to several mechanisms. Recent studies have contested the validity of heart rate variability (HRV) analysis on sympathetic activity measurement. But the plethora of HRV metrics may provide meaningful insights, particularly if linked with cardiovascular and autonomic nervous system parameters. However, the population-specific nature of HRV and cardiorespiratory response to altitude hypoxia are still missing. Six Italian trekkers and six Nepalese porters completed 300 km of a Himalayan trek. The ECG analysis was conducted at baseline, and before (*bBC*) and after (*aBC*) the high-altitude (HA) circuit. Urine was collected before and after the expedition in Italians, for assessing catecholamines. Heart rate increased with altitude significantly (*p* < 0.001) in the Italian group; systolic (*p* = 0.030) and diastolic (*p* = 0.012) blood pressure, and mean arterial pressure (*p* = 0.004) increased with altitude. Instead, pulse pressure did not change, although the Nepalese group showed lower baseline values than the Italians. As expected, peripheral oxygen saturation decreased with altitude (*p* < 0.001), independently of the ethnic groups. Nepalese had a higher respiratory rate (*p* = 0.007), independent of altitude. The cardiac vagal index increased at altitude, from baseline to *bBC* (*p* = 0.008). Higuchi fractal dimension (HFD) showed higher basal values in the Nepalese group (*p* = 0.041), and a tendency for the highest values at *bBC*. Regarding the urinary catecholamine response, exposure to HA increased urinary levels, particularly of norepinephrine (*p* = 0.005, *d* = 1.623). Our findings suggest a better cardiovascular resilience of the Nepalese group when compared with Italians, which might be due to an intrinsic adaptation to HA, resulting from their job.

## Introduction

The occurrence of hypobaric hypoxia at high altitude (HA) challenges human homeostasis, providing an ecological model for measuring the physiological responses to such environmental stressors (Cerretelli, 2013; West, 2016; Moore, 2017), both in the short- and long-term (Beall, 2003; Mulliri et al., 2019). The cardiorespiratory system is massively affected by altitude exposure since low partial pressure of oxygen in arterial blood induces acute and chronic hemodynamic changes (West, 2016; Verratti et al., 2020a). Hyperventilation occurs as an essential step of acclimatization (San et al., 2013), with a hypoxia-related switch to rapid and shallow breaths, rather than an increase in tidal volume (Brinkman et al., 2020). Concerning blood pressure (BP), the acute response in lowlanders is typically increased during ascent, especially at the beginning of exposure (Calbet and Lundby, 2009). The BP increment occurs early even after exposure to moderate altitude (Torlasco et al., 2020) and persists after acclimatization (Parati et al., 2015). BP also increases as a chronic response, despite the fact that systemic O_2_ delivery improves with acclimatization (Calbet, 2003).

The higher the altitude, the higher the heart rate, both at rest and at a standardized workload (Gamboa et al., 2003; Princi et al., 2008). What subsequently generates a chronic acclimatization process is the maintenance of cardiac output, while heart rate may continue to be high and stroke volume decreases, probably due to a reduction in plasma volume (Reeves et al., 1987; Stembridge et al., 2019). The major extrinsic factor for heart rhythmicity is the autonomic nervous system (ANS), which regulates heart rate by modulating sympathetic and parasympathetic tone (Mangoni and Nargeot, 2008). In humans, during acute exposure to altitude hypoxia, a significant increase in sympathetic activity and heart rate occurs due to the carotid body and brain stem chemoreceptors activation (Marshall, 1994; Solomon, 2000).

The sympathetic branch of ANS acts on the cardiovascular system by stimulating all the four hemodynamic effectors: inotropism, chronotropism, cardiac preload, and afterload (Furnival et al., 1971; Schümann, 1983; Takahashi et al., 1993; Ullian, 1999). Plasmatic and urinary catecholamines levels increases in response to HA exposure in subjects staying for more than 1 week (Rostrup, 1998). However, combining pharmacological inhibition of sympathetic and/or parasympathetic control of the heart, Siebenmann et al. demonstrated reduced cardiac parasympathetic activity as the main mechanisms underlying the increase of resting heart rate in response to middle-term exposure to altitude hypoxia (Siebenmann et al., 2017). The same evidence was reported in rats, with a parasympathetic withdrawal after 24 h of exposure to 3,270 m above sea level (Beltrán et al., 2020). A few evidence exists about ethnic differences in the autonomic control of the cardiovascular response to altitude hypoxia; e.g., altered muscle sympathetic nerve activity and beneficial lower sympathetic vasoconstrictor activity have been suggested as beneficial hypoxic adaptations in Sherpas (Simpson et al., 2019). Cardioprotective phenotypes in Sherpas prevent them from developing apnea-induced bradyarrhythmias at altitude (Busch et al., 2018).

Despite broad historical use of HRV as a convenient surrogate of other measures to evaluate ANS activity, strong reservations have been recently claimed, especially regarding the sympathetic influence on HRV (Thomas et al., 2019). Moreover, in HRV analysis, the contextual measurement importance of the factors has been stressed along with the accurate interpretation of the diverse parameters, which can be clustered in time-domain, frequency-domain, and non-linear indexes (Shaffer and Ginsberg, 2017). Indeed, the recent development of non-linear metrics allowed to extend the methodological rationale of HRV, e.g., Gomes and colleagues (Gomes et al., 2017) reported physical exercise to acutely reduce the chaotic behavior of heart rate dynamics, through the complex Higuchi Fractal Dimension (HFD) analysis. Indeed, alternative approaches to the typical HRV investigation may add novel interpretation levels to the non-uniform alterations in the several HRV domains observed in response to altitude exposure (Verratti et al., 2019). Dhar et al. (2018) reported higher mean RR, LF (low frequency) power ms^2^, LF (normalized units: nu), and LF/HF ratio values, with lower RMSSD (root mean square of successive RR interval differences), NN50, pNN50 (percentage of successive RR intervals that differ by more than 50 ms), SD1 (Poincaré plot standard deviation perpendicular the line of identity), HF (high frequency) power ms^2^ and HF (nu) values, in acclimatized lowlanders (15–18 months residence at >3,500 m asl) compared to HAs native and sea-level residents never exposed to HAs. However, to our knowledge, no study investigated the differential response of non-habitual altitude trekkers, compared with the altitude porters, both lowland natives. Studying the possible differences in Westerner trekkers vs. Himalayan porters could be of great interest to identify, if any, the advantages of lowland native Nepalese porters in cardiac rhythms adaptations, for providing novel insights on the occupational adaptation to HA, and for defining extended models of physiology and pathophysiology related for current expeditioners.

In the broad and extensive background of cardiovascular responses to hypoxia, population-specificity of HRV, BP, and breathing response to middle-term altitude hypoxia remains to be elucidated. The present study aimed to explore the cardiorespiratory adaptive response during a Himalayan trek, comparing Westerners and Easterners using an ecological field study design. In particular, the present work aimed to focus on the physiological meaning of the several responses as a function of altitude and/or ethnicity, as depicted by the different metrics.

## Methods

### Study Design and Participants

This study was part of the “Kanchenjunga Exploration & Physiology” project, a subset of the broad project entitled “Environmentally-modulated metabolic adaptation to hypoxia in altitude natives and sea-level dwellers: from integrative to molecular (proteomics, epigenetics, and ROS) level,” approved by the Ethics Review Board of the Nepal Health Research Council (NHRC)—ref. no. 458. All study procedures were performed under the ethical standards of the 1964 Declaration of Helsinki and all its amendments (World Medical Association, 2013). All participants provided their written, informed consent to participate in the study.

**Figure 1:**
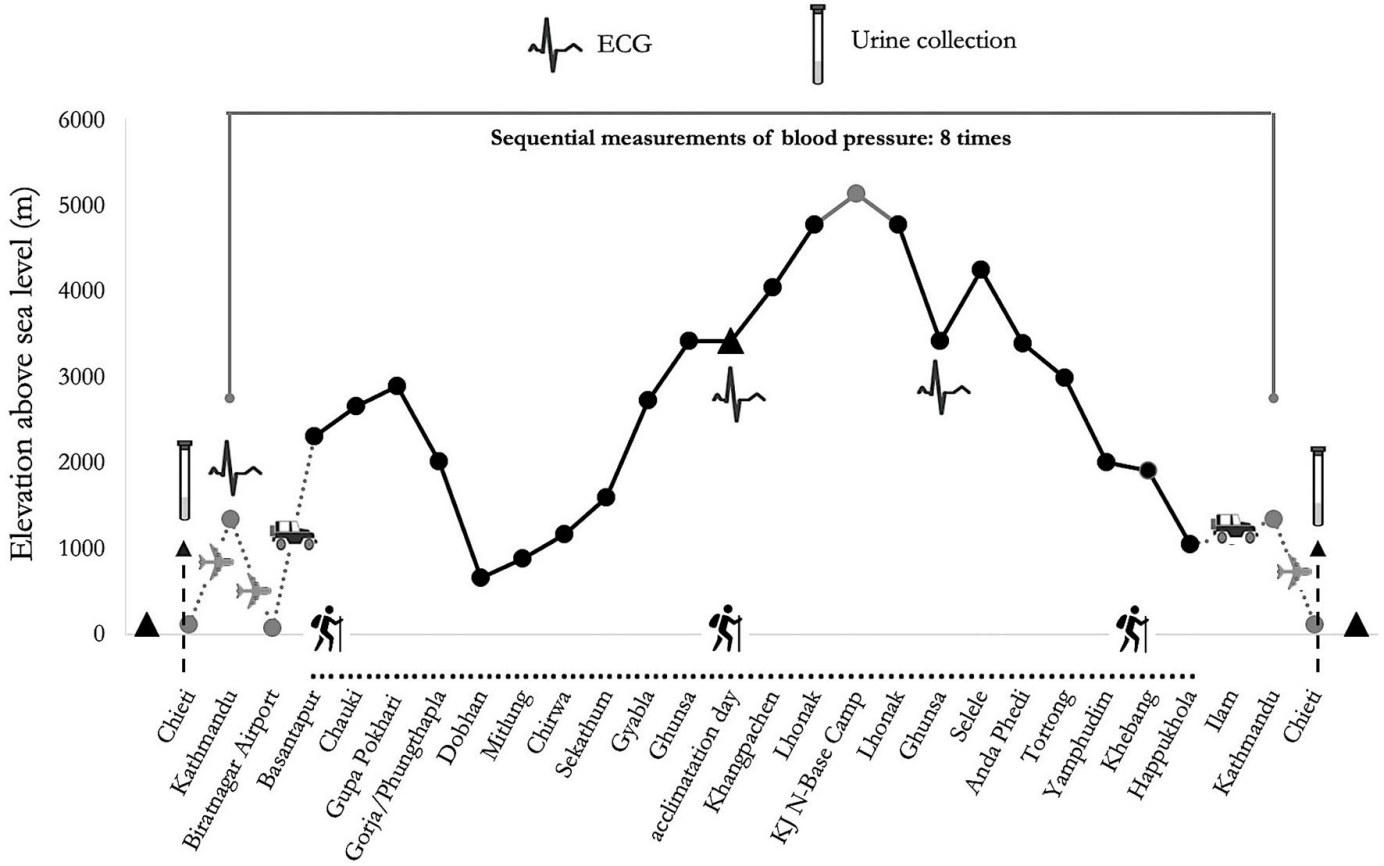
Altimetric plan and measurement time of psychological tests of “Kanchenjunga Exploration and Physiology” project.

The participants completed a combined circuit of 300 km distance (south and north base camps of Kanchenjunga), covering a daily average of about 6 h walk for about 110 h. They were exposed throughout the trek to low (500–2,000 m), moderate (2,000–3,000 m) and high (3,000–5,500 m) altitudes (Schommer and Bärtsch, 2011), along a demanding route with ascent and descent tracts covering totally over 16,000 m in altitude, in the Himalayan mountain range of eastern Nepal (Figure 1).

This research project investigated adaptive cardiovascular responses to HA exposure stressors in two experimental groups composed of six healthy Italian trekkers and six healthy Nepalese porters, both lowland dwellers. Characteristics of the two studied groups were as follows: Italians, five men, and one woman, aged 44 ± 15 years, body mass index (BMI) of 25.81 ± 3.25 Kg/m^2^; Nepalese, all men, aged 30 ± 8 years, BMI of 24.36 ± 4.70 kg/m^2^. The Italian trekkers usually live at low altitudes, and some of them reported previous HA experiences, although not in the last 3 years. The Nepalese trekkers habitually live at low altitudes and reported frequent exposure to HAs, with a working experience of 2-to-5 similar expeditions per year in the last 3 years.

The expedition was continuously supervised by an expert medical doctor. None of the participants suffered from AMS during the trek, neither did they report any cardiovascular nor respiratory disease. The Caucasian participants only took one acetazolamide pill of 250 mg daily, at 6 p.m. during the 2 days between the acclimatization day and the stay at the highest altitude point of the expedition. However, two out of three measurements were conducted before the use, and the last measurement was conducted largely after the drug washout. No drugs were used by participants that may have affected uptake transporter proteins, metabolizing enzymes, or clearance of catecholamines. The technical requirements of the devices concerning transport, storage, and operating temperature were all met. Even though distance and difference in altitude were identical among groups, the workload was different, reflecting the typical situation in modern Himalayan expeditions: while Italian trekkers carried light loads, Nepalese porters carried heavier loads (10–30 kg), throughout the whole route. All participants underwent ECG recording, before the trek (*Pre*), at 3,427 m of altitude before the North base camp (5,143 m of altitude) circuit (*bBC*), and after that circuit (*aBC*). BP and SpO_2_ assessments were conducted eight times during the trek. From urine samples collected before (*Pre*) and after the expedition (*Post*), catecholamines concentration was assessed. Urine samples were obtained only by Italians, before and 10 days after the expedition. Unfortunately, the logistics of the current study did not allow to sample and store adequately the urine samples of the Nepalese.

### Procedures and Data Analysis

Peripheral oxygen saturation (SpO_2_) was measured using a pulse oximeter (APN-100, Contec Medical Systems Co. Ltd, China); values allowing several seconds to detect a pulse and waiting for a stable value were considered. The device measured in the range of 70–100% with an accuracy of 2%. A rubber finger SpO_2_ probe was attached over a clean and dry skin (WHO, 2011), in the morning before breakfast. All the SpO_2_ tests were performed in duplicate.

Systolic blood pressure (SBP) and diastolic blood pressure (DBP) were measured using an oscillometric device (ABPM50, Contec Medical Systems Co. Ltd, China). The devices measured in a range of 40–270 mmHg with an accuracy of 3 mmHg. SBP and DBP data were taken in a sitting position at rest, with the cuff at heart level (Whelton et al., 2018), in the morning before breakfast. Pulse pressure (PP) was calculated as a diastolic–systolic difference and mean arterial pressure (MAP) was calculated as (SBP + 2DBP) ÷ 3 (Giles and Sander, 2013). All the BP tests were performed in duplicate.

**Figure 2:**
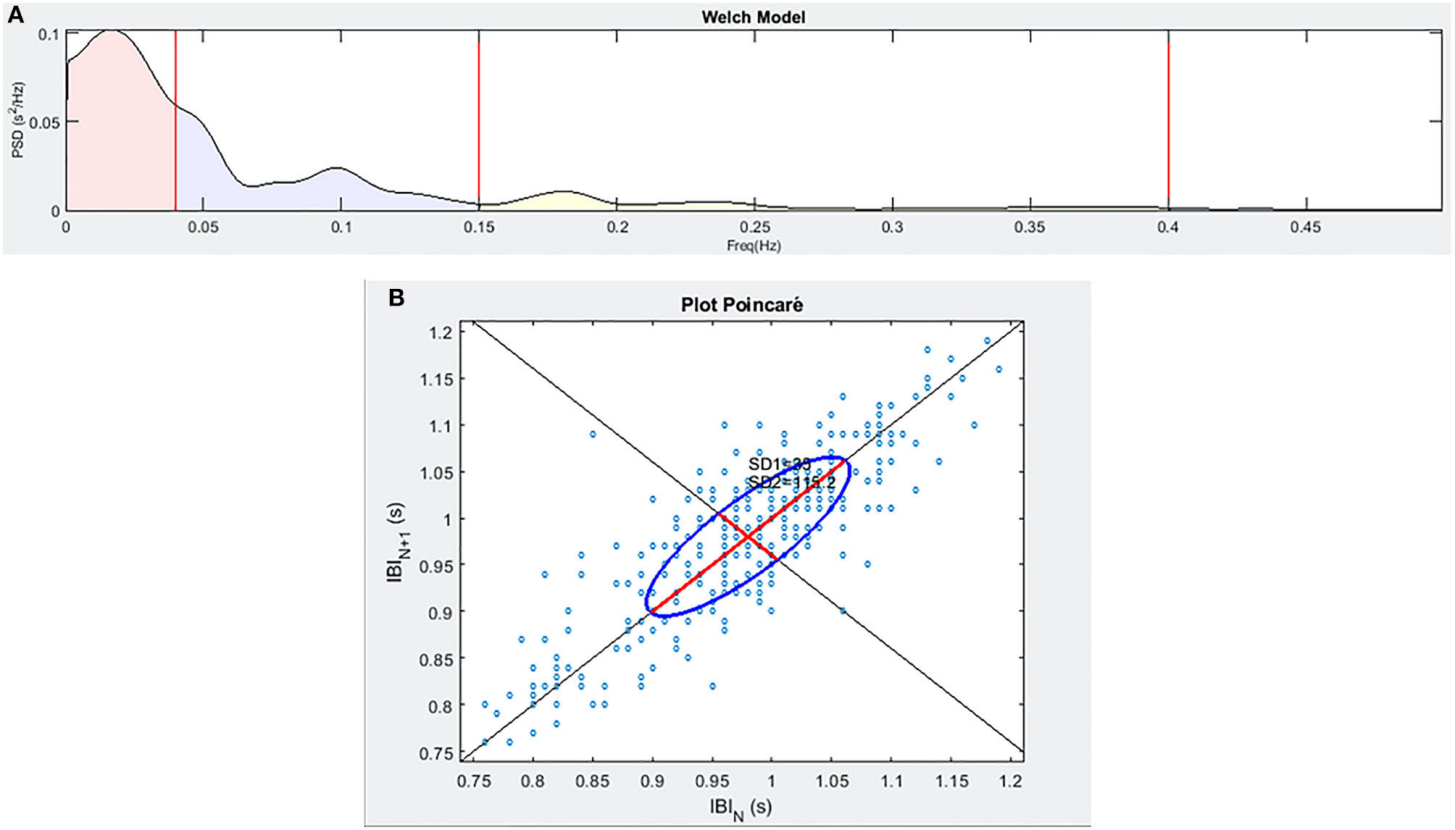
An example of the frequency spectra **(A)** and of the Poincaré plot **(B)** for a sample individual enrolled in the study.

The ECG signal was captured with the individual lying in a supine position through a single-lead Shimmer2ECG sensor (Shimmer Sensing, Dublin, Ireland) with a sampling frequency of 500 Hz to allow the estimation of the relevant HRV features according to the guidelines (Malik, 1996) and taking advantage of the Einthoven I derivative. From 5 min-ECG recording at rest, we extracted the tachogram by the Pan-Tompkins algorithm (Pan and Tompkins, 1985) and we evaluated HRV by the mean of time-domain (SDNN and pNN50), frequency-domain (absolute LF and HF power, and the normalized counterparts, computed taking into account the whole Power Spectral Density) and non-linear parameters (CSI, CVI, and HFD). Spectral calculations (see Figure 2) were computed according to the Welch model (Welch, 1967) using 64 samples in the window, 32 to overlap, and 512 points in the frequency axis. As for frequency bands, we employed the bands 0.04–0.15 Hz for LF and 0.15–0.4 Hz for HF, according to the literature (Shaffer and Ginsberg, 2017), which was also used as a reference to extrapolate considerations about short-term HRV applicability.

The sensor was attached to the body of the volunteer through a fitness-like chest strap (Polar Electro Oy, Kempele, Finland), avoiding discomfort for the individuals tested.

### Catecholamines Dosage

The obtained first-voided urinary samples were immediately stored and transferred in ice (−20°C) and subsequently analyzed frozen in liquid nitrogen for transport until analyses were carried out in Italy in the Department of Pharmacy Laboratory, University “G. d'Annunzio” of Chieti. One milliliter urine samples were stirred for 1 min using a vortex mixer, centrifuged at 13,000 *g* for 10 min to remove sediments, and finally filtered through Millipore 0.25 μm nylon filters. This was followed by HPLC-EC assay of NE and E and HPLC-UV assessment of creatinine. The detailed protocols are described below. Urinary creatinine levels were determined as previously reported (Verratti et al., 2020a,b). The HPLC apparatus consisted of a Jasco (Tokyo, Japan) PU-2080 chromatographic pump and a Jasco MD-2010 Plus absorbance detector. Integration was performed by Jasco Borwin Chromatography software, version 1.5. The chromatographic separation was performed by isocratic elution on the GraceSmart reverse phase column (C18, 150 mm × 4.6 mm i.d., 5 μm). The mobile phase was (1:99, v/v) acetonitrile and 25 mM pH 5.00 phosphate buffer containing octanesulfonic acid 10 mM and triethylamine 0.03%, v/v. The flow rate was 1.0 ml/min, and the samples were manually injected through a 20 μl loop. Creatinine analyses were performed by two hundred-fold diluting urinary samples before injection and monitoring absorbance at 235 nm. Creatinine peaks were identified by comparison with pure standard retention time, while their concentrations in the urinary samples were calculated by linear regression curve (y = bx + m) obtained with the standard. The standard stock solution of the solution of creatinine (2 mg/ml) was purchased from Alexis Biochemicals, San Diego, CA, USA. The stock solution was stored at 4°C. Work solutions (20–200 μg/ml) were daily obtained, progressively diluting stock solutions in the mobile phase. NE and E levels were analyzed through an HPLC apparatus consisting of a Jasco (Tokyo, Japan) PU-2080 chromatographic pump and an ESA (Chelmsford, MA, USA) Coulochem III coulometric detector, equipped with a microdialysis cell (ESA-5014b) porous graphite working electrode and a solid-state palladium reference electrode. The experimental conditions for biogenic amine identification and quantification were selected as follows. The analytical cell was set at −0.150 V for detector 1 and at +0.300 V for detector 2, with a range of 100 nA. The chromatograms were monitored at the analytical detector 2. Integration was performed by Jasco Borwin Chromatography software version 1.5. The chromatographic separation was performed by isocratic elution on a Phenomenex Kinetex reverse phase column (C18, 150 × 4.6 mm i.d., 2.6 μm). Regarding the separation of NE and E, the mobile phase was (10:90, v/v) acetonitrile and 75 mM pH 3.00 phosphate buffer containing octanesulfonic acid 1.8 mM, EDTA 30 μM, and triethylamine 0.015%, v/v. The flow rate was 0.6 ml/min, and the samples were manually injected through a 20 μl loop. Neurotransmitter peaks were identified by comparison with the retention time of pure standard. Neurotransmitter concentrations in the samples were calculated by linear regression curve (*y* = *bx* + *m*) obtained with the standard. The standard stock solutions of NE and E at 2 mg/ml were prepared in bidistilled water containing 0.004% EDTA and 0.010% sodium bisulfite. The stock solutions were stored at 4°C. Work solutions (1.25–20.00 ng/ml) were obtained daily by progressively diluting the stock solutions in the mobile phase.

### Statistics

The statistical analysis of HR, BP, SpO_2_, and breathing rate was carried out using the R-based open-source software Jamovi Version 1.2.27.0 (retrieved from https://www.jamovi.org). Graphs were created using the GraphPad Prism Version 5.01 (GraphPad Software, La Jolla, USA). The assumption check was based on Shapiro–Wilk's test for normality, Levene's tests for homoscedasticity and Q–Q plots. General linear mixed model (GLMM) was used to test altitude × ethnicity comparison, with residual maximum likelihood (REML) estimator, AIC and BIC as model fit measures, the Satterthwaite method for degrees of freedom, and participants as a random variable; the random effect was tested by likelihood ratio test (LRT). Marginal *R*^2^ and conditional *R*^2^ were reported, and partial eta squared ($\eta_{\text{p}}^{2}$) and partial omega squared ($\omega_{\text{p}}^{2}$) were calculated as measures of effect size (Fritz et al., 2012). Experimental data of urine NE and E were analyzed through paired sample Student's *t*-test; considering the small sample size, Cohen's *d* was adjusted to unbiased Cohen's *d* (*d*_unb_) (Fritz et al., 2012).

Concerning the ECG signal, a normality test (Shapiro–Wilk's test) was conducted prior to the overall analysis. Given the results obtained with this investigation (significant deviation from the normality), we performed non-parametric tests for the various features, including Friedman's Test with Wilcoxon *post-hoc* correction for the comparison between the different phases within the same group, or Mann–Whitney's test for comparing the data between the two different cohorts included in the investigation.

## Results

Heart rate increased with altitude; this trend was particularly evident for Italians, who also had lower overall values than Nepalese; SBP and DBP increased with altitude, as well as MAP, without significant differences by ethnicity. Instead, PP did not change, but Nepalese had lower overall values than Italians. SpO_2_ decreased with altitude, without differences in the ethnic comparison. Surprisingly, breath frequency was overall lower in Nepalese, and statistical analysis failed to demonstrate a significant response to HA exposure (see Table 1). Among the several measurements, SpO_2_ represented the best parameter to identify the response to HA, considering the greatest effect size (see Supplementary Materials) and the lowest model fit measures (AIC and BIC). In all the models, individuality played a significant role in response to HA, particularly for BP parameters.

**Table 1:** Cardiorespiratory results, clustered by altitude, and ethnic group.

**Altitude**	**Group**	**HR** **(bpm)**	**SpO_2_** **(%)**	**BR** **(bpm)**	**SBP** **(mmHg)**	**DBP** **(mmHg)**	**PP** **(mmHg)**	**MAP** **(mmHg)**
***1450m***	*Ita*	58 ± 8	98 ± 1	12 ± 4	129 ± 8	77 ± 8	52 ± 6	95 ± 8
	*Nep*	76 ± 6	96 ± 1	16 ± 4	129 ± 10	86 ± 12	43 ± 7	100 ± 11
***2316m***	*Ita*	59 ± 5	95 ± 2	12 ± 3	137 ± 13	87 ± 7	50 ± 8	103 ± 9
	*Nep*	70 ± 11	95 ± 2	18 ± 3	132 ± 18	87 ± 14	45 ± 10	102 ± 15
***2895m***	*Ita*	67 ± 8	94 ± 3	13 ± 5	131 ± 7	83 ± 9	48 ± 9	99 ± 7
	*Nep*	77 ± 12	95 ± 3	18 ± 8	137 ± 15	94 ± 13	43 ± 8	108 ± 14
***1173m***	*Ita*	62 ± 9	98 ± 1	13 ± 1	125 ± 3	81 ± 7	43 ± 5	96 ± 5
	*Nep*	76 ± 15	97 ± 2	17 ± 4	129 ± 9	86 ± 9	43 ± 6	100 ± 9
***3400m***	*Ita*	67 ± 11	92 ± 3	15 ± 2	132 ± 9	84 ± 6	48 ± 9	100 ± 6
	*Nep*	71 ± 8	92 ± 1	17 ± 4	127 ± 13	89 ± 15	38 ± 8	102 ± 14
***4750m***	*Ita*	74 ± 9	86 ± 4	15 ± 6	136 ± 10	87 v 7	49 ± 7	103 ± 8
	*Nep*	80 ± 7	85 ± 3	18 ± 5	130 ± 17	90 ± 15	40 ± 5	103 ± 16
***3000m***	*Ita*	59 ± 4	95 ± 2	13 ± 2	130 ± 12	82 v 5	48 ± 7	98 ± 7
	*Nep*	68 ± 11	95 ± 2	21 ± 3	131 ± 10	87 ± 12	44 ± 6	101 ± 11
***1747m***	*Ita*	60 ± 6	97 ± 1	13 ± 3	129 ± 9	80 ± 4	49 ± 11	96 ± 3
	*Nep*	71 ± 10	96 ± 2	18 ± 3	126 ± 12	82 ± 13	44 ± 9	97 ± 12

*Ita, Italians; Nep, Nepalese; HR, heart rate; SpO_2_, peripheral oxygen saturation; BR, breathing rate; SBP, systolic blood pressure; DBP, diastolic blood pressure; PP, pulse pressure; MAP, mean arterial pressure; bpm, beats (or breaths) per minute*.

Results of HRV are summarized in Table 2. Splitting the whole population based on nationality, Italian volunteers have displayed the same trends as the whole group concerning pNN50—a trend also reported among Nepalese participants, with no difference by altitude. In contrast, significance was found in CSI decrease at mid-point, further increase at the final evaluation, and in HFD, displaying the opposite trend. The baseline comparison between Italian and Nepalese individuals displayed higher values for Nepalese volunteers concerning HFD (1.601 ± 0.036 vs. 1.553 ± 0.026, *p* = 0.041). The total power of frequency bands was significantly higher before the trek started, compared with the midpoint; therefore, the absolute power of both VLF, LF, and HF bands followed the same trend. No other features were found to be significantly different between the two ethnicities.

**Table 2:** Data extracted from the ECG signal for the whole study population.

	**Pre**	**bBC**	**aBC**	**Pre vs. bBC**	**bBC vs. aBC**
**HR (bpm)**	66.1 ± 8.7	59.9 ± 8.7	59.4 ± 6.6	n.s.	n.s.
**SDNN (ms)**	47.8 ± 24.0	74.6 ± 37.6	65.9 ± 31.5	0.010	n.s.
**pNN50 (%)**	11.6 ± 14.0	33.1 ± 26.4	27.8 ± 23.2	0.003	n.s.
**CSI**	2.72 ± 0.86	2.26 ± 1.15	2.35 ± 0.96	n.s.	n.s.
**CVI**	3.08 ± 0.40	3.51 ± 0.52	3.42 ± 0.38	0.008	n.s.
**Total power (ms^2^)**	2.27 ± 2.56	6.37 ± 5.68	3.92 ± 4.47	0.008	n.s.
**VLF power (ms^2^)**	1.06 ± 1.18	2.49 ± 2.79	1.44 ± 1.25	n.s.	n.s.
**LF power (ms^2^)**	0.66 ± 0.91	1.68 ± 1.87	1.04 ± 1.54	0.034	n.s.
**HF power (ms^2^)**	0.55 ± 0.64	2.20 ± 2.31	1.44 ± 2.13	0.010	n.s.
**nLF (n.u.)**	0.56 ± 0.16	0.45 ± 0.18	0.45 ± 0.20	n.s.	n.s.
**nHF (n.u.)**	0.44 ± 0.16	0.55 ± 0.18	0.55 ± 0.20	n.s.	n.s.
**LF/HF**	1.57 ± 0.90	1.03 ± 0.75	1.24 ± 1.31	n.s.	n.s.
**Peak (VLF) (Hz)**	0.010 ± 0.012	0.010 ± 0.008	0.008 ± 0.009	n.s.	n.s.
**Peak (LF) (Hz)**	0.088 ± 0.029	0.105 ± 0.025	0.085 ± 0.024	n.s.	n.s.
**Peak (HF) (Hz)**	0.236 ± 0.076	0.228 ± 0.051	0.247 ± 0.067	n.s.	n.s.
**HFD**	1.577 ± 0.039	1.597 ± 0.029	1.576 ± 0.032	n.s.	n.s.

*Pre, before the trek; bBC, before the base camp circuit; aBC, after the base camp circuit; HR, heart rate; SDNN, standard deviation of the normal R-to-R intervals; pNN50, percentage of normal R-to-R intervals >50 ms; CSI, cardiac sympathetic index; CVI, cardiac vagal index; LF, low-frequency spectral band; HF, high-frequency spectral band; n.u., normalized units; VLF, very low frequency; HFD, Higuchi fractal dimension; n.s., non-significant*.

**Figure 3:**
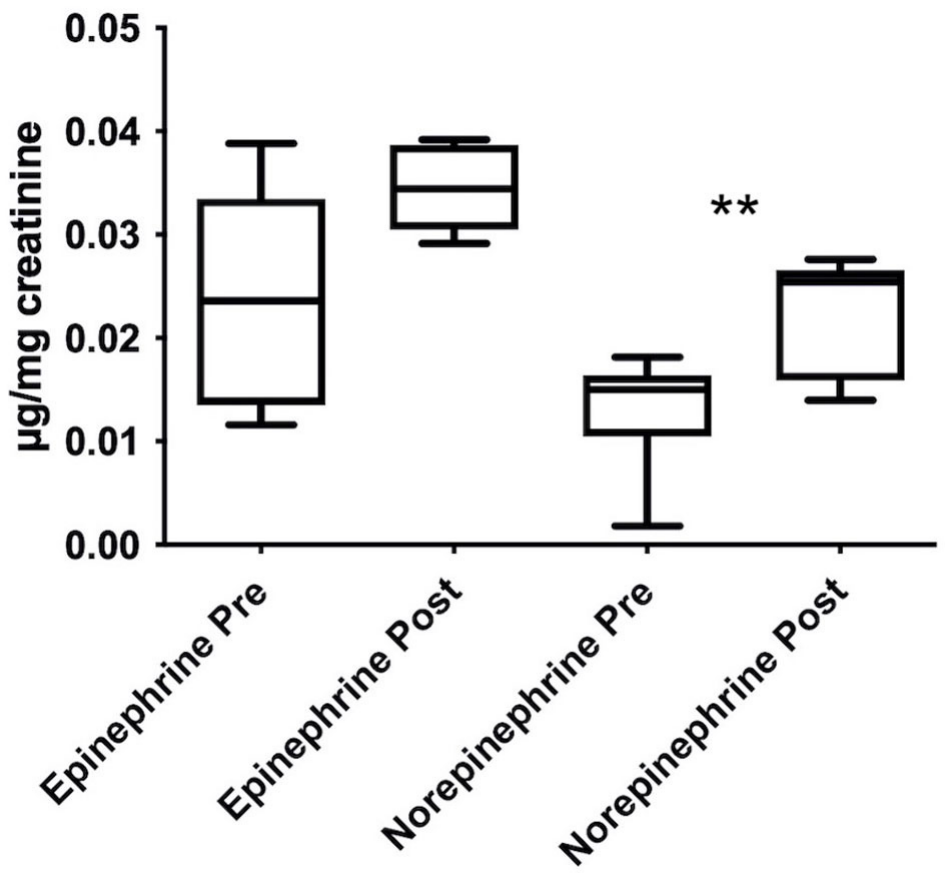
Stimulating effects induced by HA trek on urinary norepinephrine and epinephrine levels, expressed as μg/mg of creatinine and shown as boxplots. Urine samples were collected in Italians only. Data were analyzed through paired samples Student's *t*-test; **p < 0.01.

As depicted in Figure 3, the exposure to HA increased the urinary levels of epinephrine and norepinephrine, expressed as μg/mg of creatinine. This difference was significant for NE, with *t*_(5)_ = 4.719, *p* = 0.005, and *d*_unb_ = 1.623 whereas there was a strong tendency for *E*, with *t*_(5)_ = 2.382, *p* = 0.063, and *d*_unb_ = 0.819.

## Discussion

A recent massive increase in activities at HA, such as touristic sojourn, hiking, mountaineering, and sports training, raises the call for the evaluation of adaptive health consequences. In this regard, monitoring the cardiorespiratory variables emerges as a cornerstone and raises the need to use devices with adequate characteristics, i.e., compact and portable enough to perform in this harsh environment (Ridolfi et al., 2010).

Of particular importance is the assessment of a vast amount of information using simplified, low-cost, lightweight instruments, like the ECG sensor interfaced with a fitness-like chest strap. This kind of device can be carried on without a significant increase in the efforts of the carriers, but at the same time providing useful information about the ANS activation, as previously demonstrated (Billeci et al., 2019; Tonacci et al., 2019). It is worth noting that HRV metrics have been claimed as measures of ANS response, particularly regarding the sympathetic influence on heart rate and the autonomic reactivity (Thomas et al., 2019). However, logistics sometimes impairs the possibility to obtain direct measures of ANS functioning during field studies in extreme conditions. In the current work, no direct measures of ANS were conducted, but a wide set of metrics related to the cardiorespiratory system were assessed. According to the results obtained from the ECG analysis, all participants displayed likely ANS response at *bBC*. This was likely due to the higher altitude reached in this stage. This response remained sustained even at *aBC*, albeit the absolute value of SDNN did not reach the lower values displayed at the *Pre* point.

This overall trend can be affected by the higher vagal activation described by the pNN50, which could drive the overall ANS reactivity toward higher values at *bBC*. As a confirmation for this trend on the overall population, the CVI was also increased at *bBC* with respect to the Pre, with a further decrease at *aBC*, thus confirming a parasympathetic activation that occurred at the second assessment with respect to the baseline and to the third evaluation point. Conversely, limitedly to the Italian trekkers, CSI followed the opposite trend, with lower values reported at *bBC*. The higher values found for both absolute LF and HF power bands at *bBC* compared with baseline followed the higher values of total power. However, it may be misleading to use absolute power values *per se*, rather than relative values of power bands and/or integrating time-domain and non-linear values. Indeed, absolute values are greatly sensitive to artifacts and to the computational model used (Miranda Dantas et al., 2012; Stapelberg et al., 2018).

Taken together, these results might indicate a counter-intuitive autonomic behavior at HA, since several investigations have clearly demonstrated a significant sympathetic activation with the hypoxic conditions experienced at altitude (Hainsworth et al., 2007). However, it is worth noting that, in the present protocol, the *Pre* test was performed at 3,427 m, which could have driven basal measurements. Indeed, despite the response preceding the protocol start, the effects of hypoxia on the sympathetic activation within the autonomic nervous system might already appear as low as 3,000 m, especially in non-natives (Hainsworth et al., 2007).

Such effects are more likely to occur at higher altitudes. However, the concentration required to prepare the trekking might have challenged the sympathetic tone before the expedition, eventually experienced toward a higher parasympathetic activation at *bBC* with respect to the previous measurement. After the trek, the effect of physical activity on the delay of parasympathetic response, already demonstrated by others investigators (Al Haddad et al., 2012), could have determined a lack of variation with respect to the *bBC* measurement. Indeed, it can be supposed that, before the trek started, participants had greater arousal and stress with respect to *bBC*. After the base camp circuit, albeit participants were tested at the same altitude as *bBC*, the hypobaric hypoxia exposure, along with the physical conditioning, posed a high stressor on participants, determining a chronic sympathetic activation and parasympathetic de-activation, as previously reported (Siebenmann et al., 2017; Beltrán et al., 2020). Considering reduced cardiac parasympathetic activity as the main mechanisms underlying the increase of resting heart rate in response to middle-term exposure to altitude hypoxia (Siebenmann et al., 2017), and that HRV metrics could be considered more adequate in estimate parasympathetic, rather than sympathetic, dynamics (Fontolliet et al., 2018; Thomas et al., 2019), all in all, HRV can be considered effective in monitoring altitude effects on autonomic control of cardiac rhythms, when direct or more effective measures (such as microneurography) are harsh to implement.

Urinary catecholamines have been highlighted as an effective assessment of ANS role in regulating blood pressure (Missouris et al., 2016). The urinary catecholamines findings of Italians revealed the likely summatory and chronic response of the sympathetic system due to the HA trek. Indeed, consistent with our recent findings, we found an increase in urinary levels of catecholamines, thus suggesting the activation of the sympathetic nervous system following exposure to HA (Verratti et al., 2020b). Although structurally similar, catecholamines have different affinities for specific adrenergic receptors, inducing a wide range of physiological effects (Eisenhofer and Lenders, 2018). Physiologically, epinephrine produces a prolonged increase in resting energy expenditure, with increased carbohydrate oxidation (Ratheiser et al., 1998).

It has been suggested that the lipolytic activity of the skeletal muscle is increased by endogenous catecholamines *in vivo* and appears to be more responsive to epinephrine than norepinephrine stimulation (Qvisth et al., 2006). For its part, norepinephrine significantly affects blood pressure, primarily acting on alpha-adrenoreceptors in the blood vessels. In particular, urinary norepinephrine is linked with chronic alterations of blood pressure, as the higher BP, the higher urinary NE excretion (Missouris et al., 2016). Thus, a more marked increase of norepinephrine in our study indicates that BP increase after hypobaric hypoxia exposure may be due to a large extent by vasoconstriction.

Short-term exposure to hypobaric hypoxia does not increase plasma catecholamines; to be noted that, although these findings are biased by increased clearance, reduced synthesis of catecholamines during short-term hypoxia has been demonstrated (Rostrup, 1998). The time course of HRV parameters in our study, together with the chronic increase in urinary catecholamines, is in line with these findings. Therefore, we support the notion that middle (>1 week) and long-term hypobaric hypoxia exposure increases sympathetic activity (Rostrup, 1998) and consequently, blood pressure. Among the mechanisms shown to drive the cardiovascular responses to altitude hypoxia, muscle sympathetic nerve activity, sympathetic vasoconstrictor activity (Simpson et al., 2019), pulmonary arterial baroreceptors (Simpson et al., 2020), asymmetric dimethylarginine activity (Verratti et al., 2020b) have been recently pointed out.

Finally, the comparison between Italians and Nepalese individuals showed no significant differences at baseline, except for a slightly increased value for HFD in the latter. This result could demonstrate a better cardiovascular resilience (He et al., 2019) for Nepalese trekkers with respect to the Italian colleagues, possibly due to a wider experience, and consequent habit, to HAs. This phenomenon appears to be challenging in being demonstrated by “ordinary” HRV features, but non-linear, fractal measures could help retrieve possibly hidden information related to such physiological processes (Di Rienzo et al., 2010). Therefore, we suggest using non-linear measures, such as HFD (Gomolka et al., 2018), in addition to the most common ones in HRV, to evaluate hypoxia-related responses.

The result of a higher breathing rate of Nepalese porters, compared with Italian trekkers, add a novel finding to the results of Dhar and colleagues (2018), who reported a higher respiration rate of HAs native and acclimatize lowlanders (15–18 months residence at >3,500 m asl) compared with sea-level residents never exposed to HAs. As breathing rate is regulated by the brain, brainstem, respiratory muscles, lungs, airways, and blood vessels (Chourpiliadis and Bhardwaj, 2020), the ethnic difference in this summary physiological outcome, related to acute or chronic altitude exposure, may unveil novel insights on cardiorespiratory responses and adaptations.

In BP regulation at altitude, ion channels should deserve to be focused on, as they are sensitive to oxygen deprivation and play a major role in cardiac contractility and control of vasomotor tone (Shimoda and Polak, 2011). A worth investigating topic also lies on nitric oxide (NO) system, as it plays a fundamental role in cardiovascular homeostasis; NO synthases enzymes and oral nitrate-reducing bacteria supply bioactive NO (Pignatelli et al., 2020). Thus, the interplay of NOS activity, nutrition, and oral health may unveil novel insights on altitude-related hypertension. Finally, HRV has been interpreted as a global measure of the network of an organism interconnectedness and complexity, reflecting biologic rhythm variability and adaptability of the human organism (Sturmberg et al., 2015). In this vein, unveiling the different responses of HRV indexes, particularly the novel ones of non-linear analysis, will provide insights into a novel interpretation of human responses to acute and chronic environmental and physical stressors.

This study did not come without limitations, particularly related to the baseline differences between tourists and porters, and to the difference in the load carried throughout the trek. However, the ecological nature of this study did not allow to control for the baseline variables to adhere to the homogeneity of groups. These differences that may have biased the results are likely to occur in similar field studies aiming to detect the response of different populations. Therefore, further evidence is needed to extend and ameliorate the original findings herein discussed.

Considering our results, further studies may focus on the starting point of the expedition, evaluating the mood state of expeditioners, that may experience feelings such as enjoyment and emotional stresses, that are strongly linked to HRV parameters, as a sign of the well-known mind-cardiorespiratory system connection (Bernardi et al., 2001). This starting state is possibly different between the diverse groups of expeditioners (tourists, mountaineers, porters, medical doctors, and guides).

## Data Availability Statement

The raw data supporting the conclusions of this article will be made available by the authors, without undue reservation.

## Ethics Statement

The studies involving human participants were reviewed and approved by Ethics Review Board of the Nepal Health Research Council (NHRC). The patients/participants provided their written informed consent to participate in this study.

## Author Contributions

VV, AT, and DB contributed to the conception and design of the study and wrote the first draft of the manuscript. VV and DB performed the acquisition of data and samples. AT, ACh, CF, and LB performed the analyses. AT and DB performed the statistical analysis. VV, AT, DB, ACh, CF, LB, ACr, and PC wrote sections of the manuscript. All authors contributed to manuscript revision, read, and approved the submitted version.

## Conflict of Interest

The authors declare that the research was conducted in the absence of any commercial or financial relationships that could be construed as a potential conflict of interest.

## Publisher's Note

All claims expressed in this article are solely those of the authors and do not necessarily represent those of their affiliated organizations, or those of the publisher, the editors and the reviewers. Any product that may be evaluated in this article, or claim that may be made by its manufacturer, is not guaranteed or endorsed by the publisher.
