# Supplementary material for: Ethnic Differences on Cardiac Rhythms and Autonomic Nervous System Responses During a High-Altitude Trek: A Pilot Study Comparing Italian Trekkers to Nepalese Porters
Source: Front Physiol. 2021 Aug 23;12:709451. doi: 10.3389/fphys.2021.709451 (PMC8419438; doi:10.3389/fphys.2021.709451)
Supplement: Supplementary file 1 [file Table_1.DOCX]

Supplementary Material

**Supplementary Table**. Statistics of cardiorespiratory parameters' comparisons

|  | ***Altitude*** | ***Ethnicity*** | *Altitude × Ethnicity* | *R^2^ marginal* | *R^2^ conditional* | *LRT random* | *AIC* | *BIC* |
| --- | --- | --- | --- | --- | --- | --- | --- | --- |
| **HR** (bpm) | **p<.001**  **η^2^_p_=.380**  **ω^2^_p_=.318** | **p=.042**  **η^2^_p_=.326**  **ω^2^_p_=.249** | p=.227  η^2^_p_=.117  ω^2^_p_=.032 | **.330** | .715 | p<.001 | 697 | 682 |
| **SBP** (mmHg) | **p=.030**  **η^2^_p_=.183**  **ω^2^_p_=.105** | p=.888  η^2^_p_=.002  ω^2^_p_=.000 | p=.212  η^2^_p_=.118  ω^2^_p_=.034 | .083 | .692 | p<.001 | 725 | 707 |
| **DBP** (mmHg) | **p=.012**  **η^2^_p_=.212**  **ω^2^_p_=.135** | p=.297  η^2^_p_=.098  ω^2^_p_=.015 | p=.420  η^2^_p_=.089  ω^2^_p_=.002 | .135 | .703 | p<.001 | 701 | 686 |
| **PP** (mmHg) | p=.510  η^2^_p_=.079  ω^2^_p_=.000 | **p=.044**  **η^2^_p_=.318**  **ω^2^_p_=.241** | p=.586  η^2^_p_=.071  ω^2^_p_=.000 | .196 | .413 | p<.001 | 698 | 683 |
| **MAP** (mmHg) | **p=.004**  **η^2^_p_=.243**  **ω^2^_p_=.168** | p=.506  η^2^_p_=.041  ω^2^_p_=.000 | p=.226  η^2^_p_=.117  ω^2^_p_=.032 | .107 | .743 | p<.001 | 681 | 669 |
| **SpO_2_** (%) | **p<.001**  **η^2^_p_=.834**  **ω^2^_p_=.816** | p=.860  η^2^_p_=.003  ω^2^_p_=.000 | p=.425  η^2^_p_=.088  ω^2^_p_=.002 | **.735** | .807 | p<.001 | 458 | 482 |
| **BR** (bpm) | p=.318  η^2^_p_=.104  ω^2^_p_=.017 | **p=.007**  **η^2^_p_=.498**  **ω^2^_p_=.433** | p=.493  η^2^_p_=.082  ω^2^_p_=.000 | **.315** | .540 | p<.001 | 542 | 550 |
| **HR**: heart rate; **SBP**: systolic blood pressure; **DBP**: diastolic blood pressure; **PP**: pulse pressure; **MAP**: mean arterial pressure; **SpO_2_**: peripheral oxygen saturation; **BR**: breathing rate; **bpm**: beats (or breaths) per minute | | | | | | | | |
